# Study of Dengue Virus Transovarial Transmission in *Aedes* spp. in Ternate City Using Streptavidin-Biotin-Peroxidase Complex Immunohistochemistry

**DOI:** 10.3390/idr14050078

**Published:** 2022-09-28

**Authors:** Nia Kurnia, Yance Kaitana, Christina Leta Salaki, Lucia Cecilia Mandey, Josef Sem Berth Tuda, Trina Ekawati Tallei

**Affiliations:** 1 Entomology Study Program, Sam Ratulangi University, Manado 95115, Indonesia; 2 Biology Study Program, Sekolah Tinggi Keguruan dan Ilmu Pendidikan (STKIP) Kie Raha, Ternate 97716, Indonesia; 3 Department of Parasitology, Faculty of Medicine, Sam Ratulangi University, Manado 95115, Indonesia; 4 Department of Biology, Faculty of Mathematics and Natural Sciences, Sam Ratulangi University, Manado 95115, Indonesia

**Keywords:** dengue virus, transovarial transmission, immunochemistry, Ternate

## Abstract

*Aedes aegypti* is the most dominant vector in the transmission of dengue hemorrhagic fever (DHF). In addition to *Ae. aegypti*, *Ae. albopictus* is a secondary vector of the dengue virus, and both species are widespread in Indonesia. The dengue virus is transmitted from person to person through the bite of an *Aedes* spp. The vertical (transovarial) transmission of the dengue virus from infective female mosquitoes to their offspring is one of the means by which the dengue virus maintains its existence in nature. Transovarial dengue virus transmission in *Aedes* spp. mosquitoes contributes to the spread and maintenance of the dengue epidemic. This study employed a qualitative survey to detect dengue virus transovarial transmission in Ternate using the streptavidin-biotin-peroxidase complex (ISBPC) immunohistochemical test. The ISBPC examination of samples collected from the four subdistricts in Ternate revealed a positive result for transovarial transmission of dengue virus. Four *Aedes* spp., including two *Ae. aegypti* females, one *Ae. albopictus* female, and one *Ae. albopictus* male, tested positive for transovarial transmission of dengue virus in the district of North Ternate. Four *Aedes* spp., including three *Ae. aegypti* females and one *Ae. aegypti* male, were found to be positive for the transovarial transmission of dengue virus in the Central Ternate district. Seven *Aedes* spp., including five *Ae. aegypti* females, one *Ae. aegypti* male, and one *Ae. albopictus* female, tested positive for transovarial transmission of the dengue virus in the district of South Ternate city. One *Ae. aegypti* male showed positive results for transovarial transmission of dengue virus in the Ternate Island District. In this study, the transovarial transmission of the dengue virus occurred in both *Aedes* spp. female and male mosquitoes. It was demonstrated that *Aedes* spp. carry the dengue virus in their ovaries and can pass it on to their offspring. As a result, the cycle of passing the dengue virus on to local mosquito populations in the city of Ternate is not going to end just yet.

## 1. Introduction

Dengue hemorrhagic fever (DHF) is an arbovirus disease caused by the dengue virus, which belongs to the Flaviviridae family [1]. *Aedes aegypti* is by far the most significant agent in the dissemination of DHF, whereas *Ae. albopictus* is the second, less effective, vector [2]. The mosquito transmits the dengue virus from person to person through its bite. The mosquito becomes infectious by sucking the blood of an infected person and transmitting the virus to another [3].

The mosquitoes are able to continue their life cycle despite exposure to the dengue virus because the virus does not cause any cytopathic effect [4]. Two mechanisms allow the dengue virus to maintain its existence in nature: horizontal transmission between viremia vertebrates transmitted by the mosquitoes, and vertical (transovarial) transmission from infected female mosquitoes to the next generation [5]. 

Initially, it was believed that transovarial transmission of the dengue virus did not play a role in the epidemiology of dengue transmission. Recent evidence suggests that transovarial transmission of the dengue virus in *Ae. aegypti* contributes to the spread and maintenance of the dengue epidemic [6]. Immunocytochemical techniques can be used to determine whether or not transovarial transmission is present [7]. Dengue virus can also be detected using other methods, including immunofluorescence, immune-peroxidase, and polymerase chain reaction (PCR) [8,9]. 

Ternate, in North Maluku province, is one of the cities in Indonesia where dengue fever is currently endemic. Cases of DHF occur nearly every year, particularly in the city of Ternate. Detection of transovarial transmission of dengue virus in *Aedes* spp. can serve as an alternative early warning system in vector control for predicting human dengue virus transmission. Since there are no data available regarding the transovarial transmission of the dengue virus in this city, it is imperative that research be conducted into the mechanism. An investigation into the possibility of transovarial transmission was carried out using the streptavidin-biotin-peroxidase complex (ISBPC) immunohistochemical test. It is hoped that up-to-date information can be uncovered through this study, which can be used as a guide for bolstering effective and sustainable vector control management in lowering dengue fever-related morbidity and mortality.

## 2. Materials and Methods

### 2.1. Mosquito Imago Preparation

This study was conducted in Ternate City, which is located in the province of North Maluku, Indonesia. The larvae used in this study were in the third or fourth instar. Larvae were collected from four subdistricts in an endemic village of DHF in the city of Ternate. The larvae were bred until imago stage. The collected larvae were placed on a tray. The larvae were given 20 mg of dog food per day and this was increased according to the number and size of the larvae. Every day, dead larvae were removed using a pipette. Individual larvae were scooped or captured with a pipette and transferred to another tray to replace the dirty water in the original tray. When the larvae matured into pupae, they were placed in a plastic tray filled with water before being transferred to the mosquito cage for rearing. Therefore, if the pupa matured into an adult mosquito, it was already contained in the mosquito cage. The dead mosquitoes in the cages were removed daily with an aspirator and the cages were cleaned weekly with a wet cloth. Seven-day-old imagoes were collected using an aspirator and put into a jar for storage.

### 2.2. Immunochemical Test

The ISBPC immunohistochemical assay was utilized to examine mosquito head press preparations for the presence of the dengue virus. The examination was carried out at the Parasitology Laboratory of the Faculty of Medicine, Sam Ratulangi University. The head of the adult mosquito was separated from the body and then pressed on the object glass. Cold methanol was used to fix the preparations on the object glass at 20 °C for 3–5 min. The preparations were briefly washed under running water before being treated with a peroxidase blocking solution to eliminate endogenous peroxidase activity for 10 min at room temperature. The prepared commercial monoclonal antibody (DSSC7, diluted to 1:200 in phosphate-buffered saline (PBS) pH 7.4) was added at a maximum of 100 µL per preparation (adjusted until all parts were flooded) and incubated for 24 h in the refrigerator. The preparations were then washed for 5 min with PBS. After adding up to 100 µL of biotinylated universal secondary antibody per preparation, they were incubated at room temperature (25 °C) for 10 min. The preparations were washed with PBS for an additional five minutes. They were then incubated for 10 min with a ready-to-use streptavidin/peroxidase complex reagent. Following that, the preparations were washed with PBS for 5 min. The preparations were examined using a microscope with a 400× magnification. The head of an uninfected mosquito was used as a control.

## 3. Results

**Figure 1:**
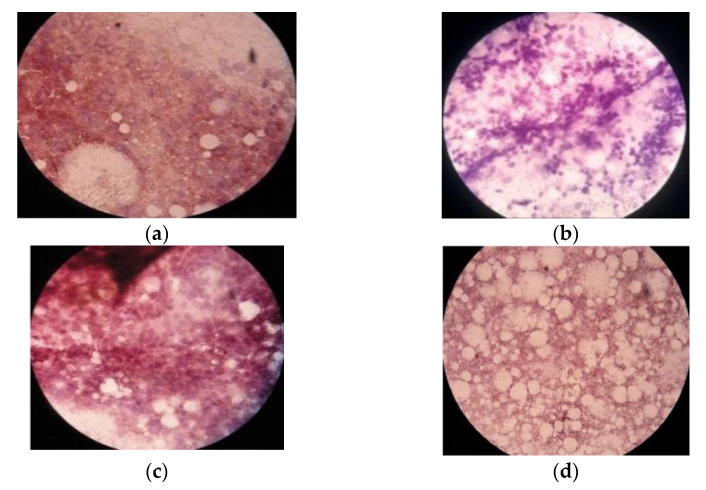
The results of microscopic preparations of the ISBPC assay: (**a**) positive control; (**b**) negative control; (**c**) positive result of *Ae. Aegypti*; (**d**) negative result of *Ae. Albopictus*. The presence of antigen was indicated by the brown color of the granules and cytoplasm of hemocytes from head squash preparations. A negative result is indicated by the absence of visible brown or blue color.

Immunohistochemical imaging of microscopic preparations of *Ae. aegypti* and *Ae. albopictus* with a commercial anti-dengue monoclonal antibody revealed immunoreaction of the preparation (Figure 1). ISBPC examination of samples collected from four subdistricts in Ternate revealed a positive result for transovarial transmission of dengue virus. The results of the examination are summarized in Table 1. If antigen is present, the granules and cytoplasm of hemocytes in preparations are brown in color that appears dispersed throughout brain tissue, whereas the majority of samples exhibited negative immunoreaction, as indicated by blue and purple granules. This conforms to the principle of steps in the immunocytochemical method of examination. To eliminate endogenous peroxidase activity, the peroxidase blocking solution was soaked and then washed in PBS. Soaking prevents the formation of non-specific bonds, which could interfere with the products of the reaction. The target antigen was then attached to the primary antibody. The addition of a biotin-labeled secondary antibody acts as a link between the primary antibody and the streptavidin-peroxidase conjugate. The conjugate is responsible for binding biotin residues. The addition of peroxidase enzyme and chromogen-substrate solution results in a change in color. The peroxidase enzyme will catalyze the substrate, hydrogen peroxide, and transform the chromogen into brown deposits, indicating the presence of antigen. 

In the North Ternate district, 22 *Aedes* spp. were obtained as a result of rearing. From these results there were ten *Ae. aegypti* females, one of which was positive for transovarial transmission of the dengue virus. Meanwhile, the rearing outcomes yielded one *Ae. albopictus* female, and the specimen tested positive for transovarial transmission. A total of nine *Ae. albopictus* males were found, but only one of them tested positive for transovarial transmission. On the other hand, no *Ae. aegypti* male tested positive for transovarial transmission.

There were fourteen *Aedes* spp. from rearing results in the Central Ternate district, including nine *Ae. aegypti* females, three of which tested positive for transovarial transmission. Four *Ae. aegypti* males were identified based on these results, one of which was positive for transovarial transmission. There were no individuals of *Ae. albopictus*, either male or female, that showed any indication of transovarial transmission.

A total of fifteen *Aedes* spp. individuals were obtained through rearing from South Ternate. Twelve of them were *Ae. aegypti* females, of which five demonstrated transovarial transmission. Additionally, two *Ae. aegypti* male were obtained, one of which tested positive for transovarial transmission. 

The rearing results from Ternate Island showed the presence of seven *Aedes* spp. The only positive result of transovarial transmission was in one *Ae. aegypti* male. Transovarial transmission was not observed in *Ae. aegypti* and *Ae. albopictus* females or *Ae. albopictus* males. Of the four districts in Ternate City, the most positive samples of transovarial transmission of dengue virus were found in the district of South Ternate.

## 4. Discussion

Transovarial transmission of the dengue virus was demonstrated in *Ae. aegypti* and *Ae. albopictus* mosquitoes in this study. The transovarial transmission of the dengue virus in *Aedes* mosquitoes has been the subject of research as an important mechanism for the maintenance of the virus in nature. This mechanism may also play a role in disease outbreaks and epidemics [6]. Previous research has shown that the district of South Ternate has the highest transovarial transmission rates. With 78,300 inhabitants, this district is the most populous in the city [10]. The finding of transovarial transmission in this study confirms the previous opinion which stated that female mosquitoes infected with dengue virus in their ovaries can transmit the virus to their offspring transovarially [7,11]. However, only a small number of studies have used transovarial transmission and monitoring of viral vectors as tools for control and surveillance. There is also evidence that the Zika virus infects mosquito ovaries and is vertically transmitted at a low rate [12]. 

The findings regarding the transovarial transmission of virus show that the presence of the dengue virus in nature is maintained vertically and without necessarily infecting the host. The mechanism of transovarial transmission takes place when the virus is transferred into the egg during fertilization through the oviduct during embryogenesis. This results in infected eggs, producing infectious larvae which will later become mosquitoes with infection rates that are greater than 80% [13]. 

The dengue virus is transmitted from infected female mosquitoes to their offspring through three different mechanisms: (1) the infectious female mosquito feeds on the blood of the host by biting them and sucking their blood. This ripens the eggs and allows the virus to reproduce (replicate) in the mid-gut of the larvae, which makes them infectious [14]; (2) non-infective female mosquitoes mate with infective male mosquitoes causing infection of the female mosquitoes [15]; and (3) the ovarian tissue of female mosquitos is infected with a virus that can be transmitted to the next generation [16].

The dengue virus will propagate in the embryos inside the eggs of an infective-gravid female mosquito. Infected eggs could possibly endure for months [17]. After hatching, the virus employs the larvae that will eventually become imago as a living medium for its propagation. These mosquitoes can infect humans with the dengue virus when they bite and draw blood [18]. In *Ae. aegypti* that survived to the seventh generation, transovarial dengue virus infection was still detected (12.6%); infection in female mosquitoes (14.8%) was higher than in male mosquitoes (10%) [19].

It was initially believed that transovarial transmission of the dengue virus played no role in the epidemiology of dengue. One study indicates that transovarial transmission of the dengue virus in *Ae. albopictus* was detected 7–41 days prior to the first reported clinical case of DHF in human [20]. If the presence of dengue virus transovarial infection in *Aedes* spp. persists, the chain of dengue virus transmission in mosquitoes will continue. Transovarial dengue virus infection in *Ae. aegypti* and *Ae. albopictus* can be found throughout the year regardless of the season. It is obvious that transovarial transmission can be viewed as a reservoir for virus survival in nature.

In this study, the transovarial transmission of the dengue virus occurred in both male and female *Aedes* spp. mosquitoes. Although male mosquitoes do not feed on the blood of infected humans, they can still become infected with the dengue virus through transmission from infected female parent. It is possible for a dengue virus infection to be passed on to the next generation if an infected male mosquito mates with a dengue virus-negative female mosquito. This will result in the generation of dengue virus-positive offspring. It was demonstrated that *Aedes* spp. carry the dengue virus in their ovaries and can pass it on to their offspring. If the *Aedes* spp. mosquito population is not completely eradicated, the cycle of transmission of the dengue virus from humans to mosquitoes in the city of Ternate will continue unabated.

## 5. Conclusions

This study provided conclusive evidence that the transovarial transmission of the dengue virus occurs in *Ae. aegypti* and *Ae. albopictus*, both of which are known to transmit dengue fever in Ternate, Indonesia. The district of South Ternate exhibited the highest levels of transovarial transmission. In Ternate City, transovarial transmission was discovered not only in female but also in male *Aedes* mosquitoes. The existence of transovarial transmission of dengue virus by *Aedes* spp. mosquitoes suggests that the mosquito-borne transmission of dengue virus in Ternate City is continuing. This transovarial transmission contributes to the spread and maintenance of the dengue virus in nature. This is because mosquitoes that are infected through transovarial transmission have the ability to transmit the dengue virus, which is present in their salivary glands, when they bite healthy humans. Therefore, it is recommended that the management of mosquito vector eradication in endemic areas should be carried out comprehensively by taking into account the potential for transovarial transmission of the dengue virus.

## Figures and Tables

**Table 1:** ISBPC examination results of *Aedes* spp. specimen in four Ternate City districts.

Location	Mosquito Species	Number of Mosquitoes Identified	ISBPC Results	
			Positive	Negative
North Ternate	*Ae. aegypti* female	10	2	8
	*Ae. aegypti* male	2	0	2
	*Ae. albopictus* female	1	1	0
	*Ae. albopictus* male	9	1	8
Central Ternate	*Ae. aegypti* female	9	3	6
	*Ae. aegypti* male	4	1	3
	*Ae. albopictus* female	1	0	1
	*Ae. albopictus* male	0	0	0
South Ternate	*Ae. aegypti* female	12	5	7
	*Ae. aegypti* male	2	1	1
	*Ae. albopictus* female	1	0	1
	*Ae. albopictus* male	0	0	0
Ternate Island	*Ae. aegypti* female	3	0	3
	*Ae. aegypti* male	4	1	3
	*Ae. albopictus* female	1	0	1
	*Ae. albopictus* male	2	0	2

## Data Availability

Not applicable.

## References

[1] Murugesan A., Manoharan M. (2020). Dengue Virus. Emerg. Reemerg. Viral Pathogens.

[2] Murray N.E.A., Quam M.B., Wilder-Smith A. (2013). Epidemiology of Dengue: Past, Present and Future Prospects. Clin. Epidemiol..

[3] Boulaaras S., Jan R., Khan A., Ahsan M. (2022). Dynamical Analysis of the Transmission of Dengue Fever via Caputo-Fabrizio Fractional Derivative. Chaos Solitons Fractals X.

[4] White A.V., Fan M., Mazzara J.M., Roper R.L., Richards S.L. (2021). Mosquito-Infecting Virus Espirito Santo Virus Inhibits Replication and Spread of Dengue Virus. J. Med. Virol..

[5] Rosen L., Shroyer D.A., Tesh R.B., Freier J.E., Lien J.C. (1983). Transovarial Transmission of Dengue Viruses by Mosquitoes: Aedes Albopictus and Aedes Aegypti. Am. J. Trop. Med. Hyg..

[6] da Costa C.F., Dos Passos R.A., Lima J.B.P., Roque R.A., de Souza Sampaio V., Campolina T.B., Secundino N.F.C., Pimenta P.F.P. (2017). Transovarial Transmission of DENV in Aedes Aegypti in the Amazon Basin: A Local Model of Xenomonitoring. Parasit. Vectors.

[7] Sudarmaja I.M., Swastika I.K., Diarthini L.P.E., Prasetya I.P.D., Wirawan I.M.A. (2022). Dengue Virus Transovarial Transmission Detection in Aedes Aegypti from Dengue Hemorrhagic Fever Patients’ Residences in Denpasar, Bali. Vet. World.

[8] Gurukumar K.R., Priyadarshini D., Patil J.A., Bhagat A., Singh A., Shah P.S., Cecilia D. (2009). Development of Real Time PCR for Detection and Quantitation of Dengue Viruses. Virol. J..

[9] Sucipto T.H., Ahwanah N.L.F., Churrotin S., Matake N., Kotaki T., Soegijanto S. (2016). Immunofluorescence Assay Method to Detect Dengue Virus in Paniai-Papua. AIP Conf. Proc..

[10] Tomia S., Hadi U.K., Soviana S., Retnani E. (2022). Epidemiologi Kejadian Demam Berdarah Dengue Di Kota Ternate, Maluku Utara. J. Vet..

[11] Araini D.H.W., Marsifah T., Mustangin Y., Poerwanto S.H. (2019). Detection of Transovarial Transmission of Dengue Virus in Aedes Spp. (Diptera: Culicidae) from Brontokusuman Village, Yogyakarta, Indonesia. Biodiversitas.

[12] Nag D.K., Payne A.F., Dieme C., Ciota A.T., Kramer L.D. (2021). Zika Virus Infects Aedes Aegypti Ovaries. Virology.

[13] Beaty B.J., Woodring J.L., Higgs S. (1996). Natural Cycles of Vector-Borne Pathogens. The Biology of Disease Vectors.

[14] Mukherjee D., Das S., Begum F., Mal S., Ray U. (2019). The Mosquito Immune System and the Life of Dengue Virus: What We Know and Do Not Know. Pathogens.

[15] Mavale M., Parashar D., Sudeep A., Gokhale M., Ghodke Y., Geevarghese G., Arankalle V., Mishra A.C. (2010). Venereal Transmission of Chikungunya Virus by Aedes Aegypti Mosquitoes (Diptera: Culicidae). Am. J. Trop. Med. Hyg..

[16] Sánchez-Vargas I., Harrington L.C., Doty J.B., Black W.C., Olson K.E. (2018). Demonstration of Efficient Vertical and Venereal Transmission of Dengue Virus Type-2 in a Genetically Diverse Laboratory Strain of Aedes Aegypti. PLoS Negl. Trop. Dis..

[17] Buckner E.A., Alto B.W., Lounibos L.P. (2013). Vertical Transmission of Key West Dengue-1 Virus by Aedes Aegypti and Aedes Albopictus (Diptera: Culicidae) Mosquitoes from Florida. J. Med. Entomol..

[18] Sorisi A.M.H. (2013). Transmisi Transovarial Virus Dengue Pada Nyamuk *Aedes* spp.. J. Biomedik.

[19] Joshi V., Singhi M., Chaudhary R.C. (1996). Transovarial Transmission of Dengue 3 Virus by Aedes Aegypti. Trans. R. Soc. Trop. Med. Hyg..

[20] Lee H.L., Rohani A. (2005). Transovarial Transmission of Dengue Virus in Aedes Aegypti and Aedes Albopictus in Relation to Dengue Outbreak in an Urban Area in Malaysia. Dengue Bull..

